# Risk of ischaemic stroke associated with antiepileptic drugs: a population-based case-control study in Catalonia

**DOI:** 10.1186/s12883-021-02237-1

**Published:** 2021-05-24

**Authors:** Maria Giner-Soriano, Josep Ramon Marsal, Ainhoa Gomez-Lumbreras, Rosa Morros

**Affiliations:** 1 Fundació Institut Universitari per a la recerca a l’Atenció Primària de Salut Jordi Gol i Gurina (IDIAPJGol), Gran Via de les Corts Catalanes 587, àtic, 08007 Barcelona, Spain; 2 Universitat Autònoma de Barcelona, Cerdanyola del Vallès Bellaterra, Spain; 3 Unitat d’Epidemiologia, Servei de Cardiología, Hospital Universitari Vall d’Hebron, Universitat Autònoma de Barcelona, CIBERESP, Barcelona, Spain; 4 Facultat de Medicina, Universitat de Girona, Girona, Spain; 5 Institut Català de la Salut, Barcelona, Spain

**Keywords:** Antiepileptic drugs, Stroke, Drug exposure, Electronic health records, Primary healthcare

## Abstract

**Background:**

Cerebrovascular disorders have occurred more frequently in some Central Nervous System (CNS) disorders, such as epilepsy. Some CNS drugs have been associated with increased stroke risk. Our aim was to estimate the risk of ischaemic stroke in patients exposed to antiepileptic drugs (AED).

**Methods:**

Population-based matched case-control study using SIDIAP database, based in electronic health records from primary healthcare from Catalonia, Spain. Cases were those patients with a registered diagnosis of first stroke during 2009–2014. Up to 10 controls were selected for each case and matched by sex, age, and geographic area and without a prior diagnosis of stroke. We considered global drug exposure to AED, past and current exposure and exposure in monotherapy to each AED.

**Results:**

2,865 cases and 19,406 controls were exposed to AED during the study period. Global exposure to levetiracetam [(OR_adj_3.3, CI95 % 2.8-4.0)], phenytoin [OR_adj_1.5, CI95 % 1.2–41.9)], and valproic acid [(OR_adj_ 1.3, CI95 % 1.1–1.6)], showed significantly association to ischaemic stroke that was also maintained with current exposure of levetiracetam [OR_adj_4.1, CI95 % 3.3–5.2)] and valproic acid [OR_adj_1.4, CI95 % 1.1–1.9)]. Current levetiracetam monotherapy showed a very high risk of ischaemic stroke [(OR_adj_ 5.1, CI95 % 3.7–6.9)].

**Conclusions:**

Drugs used for other conditions than epilepsy (pregabalin, gabapentin) were the most used AED and both did not show a risk. Levetiracetam shows a risk for stroke even when assessed in current monotherapy. The lack of data regarding the link with diagnosis and severity in our study makes it necessary to conduct further studies to confirm or dismiss our results, focussing on levetiracetam.

**Supplementary Information:**

The online version contains supplementary material available at 10.1186/s12883-021-02237-1.

## Background

Stroke is the second leading cause of death worldwide [1]. There are four types: ischaemic stroke, primary intracerebral haemorrhage, subarachnoid haemorrhage and undefined, being the ischaemic type the most frequent one (80–85 %) in Caucasian population [2, 3]. Stroke patients are at highest risk of death in the first weeks after the event, and between 20 and 50 % die within the first month depending on type, severity, age, comorbidity and effectiveness of treatment of complications. Those who survive can remain with or without disabilities, such as loss of speech and movement, making strokes the third cause of disability [1]. Over the last decades the total number of age-standardized rates of stroke mortality have decreased meanwhile stroke survivors have made the burden of stroke Disability-Adjusted Life Year (DALY lost) increase [2].

The pooled proportional frequency of ischaemic stroke in 2000–2008 was lower in low to middle income countries than in high-income countries (67 and 82 %, respectively) [2]. In high income countries in 2000–2008, early (21 days to 1 month) case fatality was between 13 and 23 %, [2] being in Catalonia mortality rate standardized by age 29.2 deaths/100,000 inhabitants in 2016 [4].

The most important modifiable risk factors for ischaemic stroke are hypercholesterolemia, hypertension, diabetes and smoking [5, 6].

Cerebrovascular disorders have occurred more frequently in some Central Nervous System (CNS) disorders, such as epilepsy [7–10] and also some CNS drugs have been reported to increase the risk for stroke, such as antidepressants, [11] anti-dementia drugs, [12] antipsychotics [13, 14] or antiepileptic drugs (AED) [15–17]. The mechanism suggested for AED is that they would increase risk of vascular diseases by accelerating atherosclerosis; for example cytochrome P450 enzyme-inducing AED as carbamazepine increase serum levels of total cholesterol and LDL-cholesterol and lipoprotein [16]. Concurrently, AED are often administered after a stroke to treat seizures or other conditions [18–20].

As there has been an increase in the chronic use of AED, on account of its use for the treatment of diseases different from epilepsy, like chronic neuropathic pain, migraine, anxiety and as mood stabilizers, [21] we think it is relevant to confirm if the risks detected in other studies are confirmed in our population. The aim of this study was to estimate the risk of ischaemic stroke in patients exposed to AED.

## Methods

### Study design

Population-based matched case-control study using data from primary healthcare (PHC) electronic records.

### Study source

Information System for Research in Primary Care (SIDIAP) database; which contains data from 279 PHC centres from the Catalan Health Institute (ICS), covering 5.8 million people (80 % of the Catalan population) [22]. SIDIAP contains anonymized clinical information originated from different data sources: (1) ECAP (electronic health records in PHC of the ICS), including information since 2006 on sociodemographic characteristics, health conditions registered as International Classification Diseases Tenth Revision (ICD-10) [23] code, general practitioners’ (GP) prescriptions, clinical parameters and toxic habits, (2) laboratory data, (3) pharmacy invoice data corresponding to GP’s prescriptions (available since 2005) including information on all pharmaceutical products dispensed by communities pharmacies with Catalan Health System prescriptions by Anatomic Therapeutic and Chemical (ATC) [24] codes, and 4), Minimum Data Set at Hospital Discharge (CMBD-HA) [25] database for ICS hospitals, including diagnoses at hospital discharge registered as International Classification Diseases, Ninth Revision (ICD-9) codes [26].

### Selection of cases and controls

Cases were those patients ≥ 18 years old suffering a first ischaemic stroke (CMBD-HA hospital admission ICD 9th codes: 433, 434, 435, 436, 437, 438) during 2009–2014 attended in hospitals from the ICS in Catalonia, Spain. Index date was the day of hospital admission for a stroke from January 2009 to December 2014.

A total of 10 controls were selected for each case, and matched by sex, age (+/- 1 year), geographic area and without previous diagnosis of stroke; any historic record of stroke before the selection year caused the exclusion as a control. The index date for controls was the same than for their respective case.

### Variables

Age, sex, socioeconomic index (MEDEA) [27], body mass index (BMI), smoking status, alcohol intake, comorbidities, cardiovascular risk (classified as high, moderate, low or no risk), laboratory data including glomerular filtration rate (GFR) calculated per MDRD (Modification of Diet in Renal Disease), drug exposure and number of visits in PHC during the previous year to the index date.

Patients diagnosed with ischaemic heart disease and/or peripheral artery disease and/or diabetes with complications were classified with a high cardiovascular risk. Among the unclassified patients in the high category, those with uncomplicated diabetes, treatment with antidiabetic drugs or two or three of the following diagnoses: dyslipidaemia, hypertension, and smoking (active or ex-smoking) were classified as moderate cardiovascular risk. Patients who were not classified in any category were considered to be at low cardiovascular risk (divided into two groups: very low cardiovascular risk if no risk factors registered and low cardiovascular risk if one risk factor registered).

### Exposure definition

The drugs of interest were antiepileptics, N03A from the ATC classification. Information of exposure was obtained from GP’s prescriptions and pharmacy invoice data. Current comedications before index date were also collected: anticoagulants, antiplatelets, diabetes and cardiac therapy, lipid modifying agents, hormonal contraceptives, non-steroidal anti-inflammatory drugs (NSAID), analgesics, and psychotropic drugs.

As first SIDIAP registers begin in 2005, a minimum period of four years was selected in order to assess the AED exposure.

We considered drug exposure when there was a dispensation of at least three packages of an active substance of any of the study drugs (AED) during the study period. Past exposure was considered if the dispensation had taken place more than one year before the index date. Current exposure was considered if patients had at least one dispensation within the three months before the index date. Trying to avoid indication bias, patients with one dispensation within the same month of the index date were excluded for the current exposure analysis. Also, current exposure to only one AED was also ascertained as monotherapy exposure.

### Population size

For the study period SIDIAP had around 40,000 patients with a new stroke diagnosis registered. As CMBD-HA represents about a third of the SIDIAP total population (ICS hospitals and their primary care referral areas), we aimed to include approximately 12,000 cases. Assuming that the prevalence of exposure to the studied factors would be 1 % higher in cases, the available sample size would allow for detecting significant associations with OR ≥ 1.3 with a type I error of 5 % and an 80 % power.

### Statistical analysis

We conducted multivariate models of conditional logistic regression, and we calculated crude and adjusted odds ratios (OR, OR_adj_) and their 95 % confidence interval (CI 95 %). The crude model adjusted by age, sex, year of the index date and cardiovascular risk. The variables used in the adjusted model were: age, sex, year of the index date, MEDEA index, BMI, smoking status, number of visits to PHC, GFR, and diagnoses of: arthrosis, dementia, depression, diabetes, dyslipidaemia, epilepsy, fibromyalgia, hypertension, ischaemic heart disease, neuropathy, peripheral arterial disease and gastrointestinal ulcer. Co-treatments (insulin, antihypertensive drugs, NSAID…) and all study drugs included were added in this model.

We estimated the crude and adjusted effects of the current, past and global exposure to AED, and the effect of each AED for patients exposed in monotherapy.

## Results

**Fig. 1:**
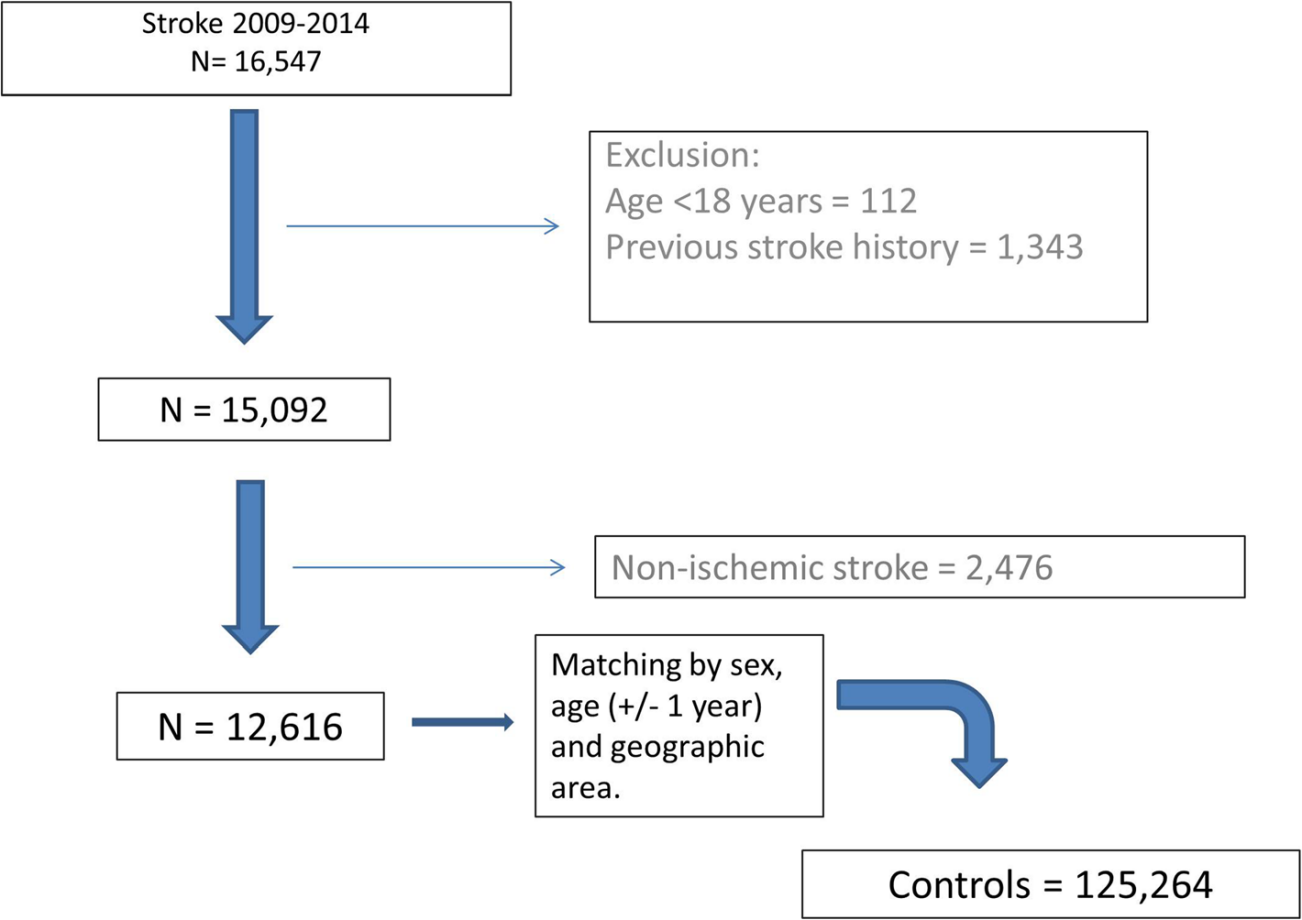
Study flowchart

A total of 137,880 patients were included in the study; they were 12,616 cases with a first ischaemic stroke during 2009–2014 matched by sex, age and geographical region with 125,264 controls with no stroke history (Fig. 1). There were 56.3 % of men and their mean age was 72.6 (SD 13.2). Their baseline sociodemographic and clinical characteristics are described in Table 1 and their comedications at baseline, in Table 2. Cases and controls were different in most baseline characteristics highlighting the neuromental illness such as dementia (cases *n* = 828, 6.6 % and controls *n* = 6075, 4.8 %,), depression (853, 6.8 6870, 5.5) and epilepsy (cases *n* = 207, 1.6 % and controls *n* = 843, 0.7 %) and also in their ischaemic heart disease history (cases *n* = 1745, 13.8 % and controls *n* = 9656, 7.7 %), all statistically significant (*p* < 0,001). Also, the comedications at baseline showed differences (cardiac therapy for cases *n* = 2448, 19.4 % and for controls *n* = 15,565, 12.4 % and insulins cases *n* = 1276, 10.1 % and controls *n* = 4796, 3.8 %) but not for antidementia drugs (cases *n* = 2020, 16.0 % and controls *n* = 20,451, 16.3 %) and coxibs and oxicams (cases *n* = 2020, 16.0 % and controls n = 20,451, 16.3 %).

**Table 1:** Sociodemographic and clinical characteristics at baseline of patients exposed to Central Nervous System drugs

**N, %**	Total *N *= 137,880	Cases *N *= 12,616	Controls *N *= 125,264	*p*-value
**Mean age, SD**	72.6, 13.2	72.9, 13.2	72.6, 13.2	0.009
**Sex, female**	60216, 43.7	5486, 43.5	54730, 43.7	0.658
**N visits in PHC**				
*Missing values*	*15165, 11.0*	*682, 5.4*	*14483, 11.6*	*<0.001*
<5	27055, 22.0	1860, 15.6	25195, 22.7	<0.001
5-9	33155, 27.0	2460, 20.6	30695, 27.7	
10-24	46191, 37.6	4837, 40.5	41354, 37.3	
≥25	16314, 13.3	2777, 23.3	13537, 12.2	
**Smoking status**				
*Missing values*	*18895, 13.7*	*1294, 10.3*	*17601, 14.1*	*<0.001*
Current smokers	16172, 13.6	2020, 17.8	14152, 13.1	<0.001
**BMI mean, SD**	28.9, 4.7	28.8, 4.9	28.9, 4.7	0.273
*Missing values*	*78443, 56.9*	*6610, 52.4*	*71833, 57.3*	*<0.001*
Obesity	21708, 36.5	2180, 36.3	19528, 36.5	<0.001
**MEDEA**				<0.001
*U*	*8431, 6.1*	*1216, 9.6*	*7215, 5.8*	
R	25909, 18.8	2343, 18.6	23566, 18.8	
U1-U3	58111, 42.1	4955, 39.3	53156, 42.4	
U4-U5	45429, 32.9	4102, 32.5	41327, 33.0	
**GFR**				
*Missing values*	*56401, 40.9*	*4186, 33.2*	*52215, 41.7*	*<0.001*
<30 mL/min/1.73m^2^	1599, 2.0	311, 3.7	1288, 1.8	<0.001
30-44	5357, 6.6	802, 9.5	4555, 6.2	
45-59	12751, 15.6	1568, 18.6	11183, 15.3	
≥60	61772, 75.8	5749, 68.2	56023, 76.7	
**Cardiovascular risk, high**	14978, 10.9	2511, 19.9	12467, 10.0	<0.001
**Comorbidities**				
Anxiety	12792, 9.3	1177, 9.3	11615, 9.3	0.836
Arthrosis	31921, 23.2	2823, 22.4	29098, 23.2	0.031
Cancer	24479, 17.8	2306, 18.3	22173, 17.7	0.106
COPD	16594, 12.0	1822, 14.4	14772, 11.8	<0.001
Dementia	6903, 5.0	828, 6.6	6075, 4.8	<0.001
Depression	7723, 5.6	853, 6.8	6870, 5.5	<0.001
Diabetes	29158, 21.1	3890, 30.8	25268, 20.2	<0.001
Dyslipidaemia	52057, 37.8	5007, 39.7	47050, 37.6	<0.001
Epilepsy	1050, 0.8	207, 1.6	843, 0.7	<0.001
Fibromyalgia	1266, 0.9	124, 1.0	1142, 0.9	<0.001
Hypertension	73250, 53.1	7626, 60.4	65624, 52.4	<0.001
Ischaemic heart disease	11401, 8.3	1745, 13.8	9656, 7.7	<0.001
Neuropathies	5067, 3.7	575, 4.6	4492, 3.6	<0.001
Peripheral artery disease	3888, 2.8	916, 7.3	2972, 2.4	<0.001
Ulcers	4274, 3.1	444, 3.5	3830, 3.1	0.005

*SD *standard deviation, *PHC *primary healthcare, *BMI *body mass index, *MEDEA *socioeconomic index, (*U *unknown urban area, *U#* urban areas), *R *rural area, *GFR *glomerular filtration rate, *COPD *chronic obstructive pulmonary disease

**Table 2:** Comedications at baseline of patients exposed to Nervous System drugs

N, %	Total *N* = 137,880	Cases *N* = 12,616	Controls *N* = 125,264	*p*-value
Acetic acid derivatives	58,208, 42.2	5652, 44.8	52,556, 42.0	< 0.001
Analgesics (metamizol and paracetamol)	109,801, 79.6	10,771, 85.4	99,030, 79.1	< 0.001
Anti-dementia agents	8907, 6.5	861, 6.8	8046, 6.4	0.080
Antidepressants	39,851, 28.9	4465, 35.4	35,386, 28.2	< 0.001
Antihypertensive agents	3774, 2.7	576, 4.6	3198, 2.6	< 0.001
Antithrombotic drugs	39,951, 29.0	8613, 68.3	31,338, 25.0	< 0.001
Antipsychotics	20,024, 14.5	2361, 18.7	17,663, 14.1	< 0.001
Anxiolytics	62,234, 45.1	6536, 51.8	55,698, 44.5	< 0.001
Beta-blockers	18,022, 13.1	2864, 22.7	15,158, 12.1	< 0.001
Blood glucose-lowering drugs	20,733, 15.0	2896, 23.0	17,837, 14.2	< 0.001
Calcium channel-blockers	18,013, 13.1	2448, 19.4	15,565, 12.4	< 0.001
Cardiac therapy	13,233, 9.6	2251, 17.8	10,982, 8.8	< 0.001
Coxibs and oxicams	22,471, 16.3	2020, 16.0	20,451, 16.3	0.369
Diuretics	28,656, 20.8	3794, 30.1	24,862, 19.8	< 0.001
Gastrointestinal tract	2505, 1.8	491, 3.9	2014, 1.6	< 0.001
Hypnotics and sedatives	23,208, 16.8	2607, 20.7	20,601, 16.4	< 0.001
Insulins	6072, 4.4	1276, 10.1	4796, 3.8	< 0.001
Lipid-modifying agents	42,897, 31.1	6119, 48.5	36,778, 29.4	< 0.001
Opioids (phentanil, buprenorphine and tramadol)	33,489, 24.3	3672, 29.1	29,817, 23.8	< 0.001
Propionic acid derivatives	93,012, 67.5	8839, 70.1	84,173, 67.2	< 0.001
Psychostimulants, ADHD-agents and nootropics	11,948, 8.7	1390, 11.0	10,558, 8.4	< 0.001
Renin-angiotensin agents	54,778, 39.7	6657, 52.8	48,121, 38.4	< 0.001
Other anti-inflammatories (glucosamine and chondroitin sulphate)	17,342, 12.6	1433, 11.4	15,909, 12.7	< 0.001

*ADHD *attention deficit hyperactivity disorder

During the study period 2,865 (22.7 %) cases were exposed to AED (ATC classification N03) and 19,406 (15.5 %) were controls. Cases were more frequently exposed to all AED than controls (Table 3), except for pregabalin (38.4 % cases vs. 44.4 % controls exposed, *p* < 0.001). The drugs with the highest proportions of patients exposed were pregabalin (8,861 39.8 %) and gabapentin (9,720 43.6 %).

**Table 3:** Exposure to antiepileptic drugs

**N, %**	Total *N* = 22,271	Cases *N* = 2,865	Controls *N *= 19,406	*p*-value
Carbamazepine exposure	1238, 5.6	169, 5.9	1069, 5.5	0.407
Current exposure	321, 1.4	52, 1.8	269, 1.4	
Past exposure	202, 0.9	32, 1.1	170, 0.9	
Monotherapy	140, 0.6	15, 0.5	125, 0.6	
Clonazepam	4675, 21.0	570, 19.9	4105, 21.2	0.128
Current	1089, 4.9	135, 4.7	954, 4.9	
Past	1019, 4.6	124, 4.3	895, 4.6	
Monotherapy	525, 2.4	52, 1.8	473, 2.4	
Gabapentin	8861, 39.8	1138, 39.7	7723, 39.8	0.951
Current	1814, 8.1	296, 10.3	1518, 7.8	
Past	1994, 9.0	244, 8.5	1750, 9,0	
Monotherapy	1060, 4.8	177, 6.2	883, 4.6	
Lamotrigine	575, 2.6	94, 3.3	481, 2.5	0.012
Current	191, 0.9	38, 1.3	153, 0.8	
Past	146, 0.7	21, 0.7	125, 0.6	
Monotherapy	72, 0.3	14, 0.5	58, 0.3	
Levetiracetam	654, 2.9	231, 8.1	423, 2.2	<0.001
Current	383, 1.7	166, 5.8	217, 1.1	
Past	62, 0.3	10, 0.3	52, 0.3	
Monotherapy	187, 0.8	90, 3.1	97, 0.5	
Oxcarbazepine	556, 2.5	83, 2.9	473, 2.5	0.137
Current	149, 0.7	27, 0.9	122, 0.6	
Past	131, 0.6	20, 0.7	111, 0.6	
Monotherapy	66, 0.3	11, 0.4	55, 0.3	
Phenobarbital	520, 2.3	85, 3.0	435, 2.2	0.018
Current	205, 0.9	39, 1.4	166, 0.9	
Past	124, 0.6	28, 1.0	96, 0.5	
Monotherapy	58, 0.3	10, 0.3	48, 0.2	
Phenytoin	610, 2.7	128, 4.5	482, 2.5	<0.001
Current	232, 1.0	49, 1.7	183, 0.9	
Past	121, 0.5	25, 0.9	96, 0.5	
Monotherapy	96, 0.4	17, 0.6	79, 0.4	
Pregabalin	9720, 43.6	1100, 38.4	8620, 44.4	<0.001
Current	1829, 8.2	239, 8.3	1590, 8.2	
Past	2586, 11.6	285, 9.9	2301, 11.9	
Monotherapy	1058, 4.8	119, 4.2	939, 4.8	
Topiramate	1088, 4.9	168, 5.9	920, 4.7	0.010
Current	176, 0.8	36, 1.3	140, 0.7	
Past	285, 1.3	45, 1.6	240, 1.2	
Monotherapy	77, 0.3	9, 0.3	68, 0.4	
Valproic acid	1037, 4.7	192, 6.7	845, 4.4	<0.001
Current	367, 1.6	92, 3.2	275, 1.4	
Past	214, 1.0	28, 1.0	186, 1.0	
Monotherapy	175, 0.8	39, 1.4	136, 0.7	

**Fig. 2:**
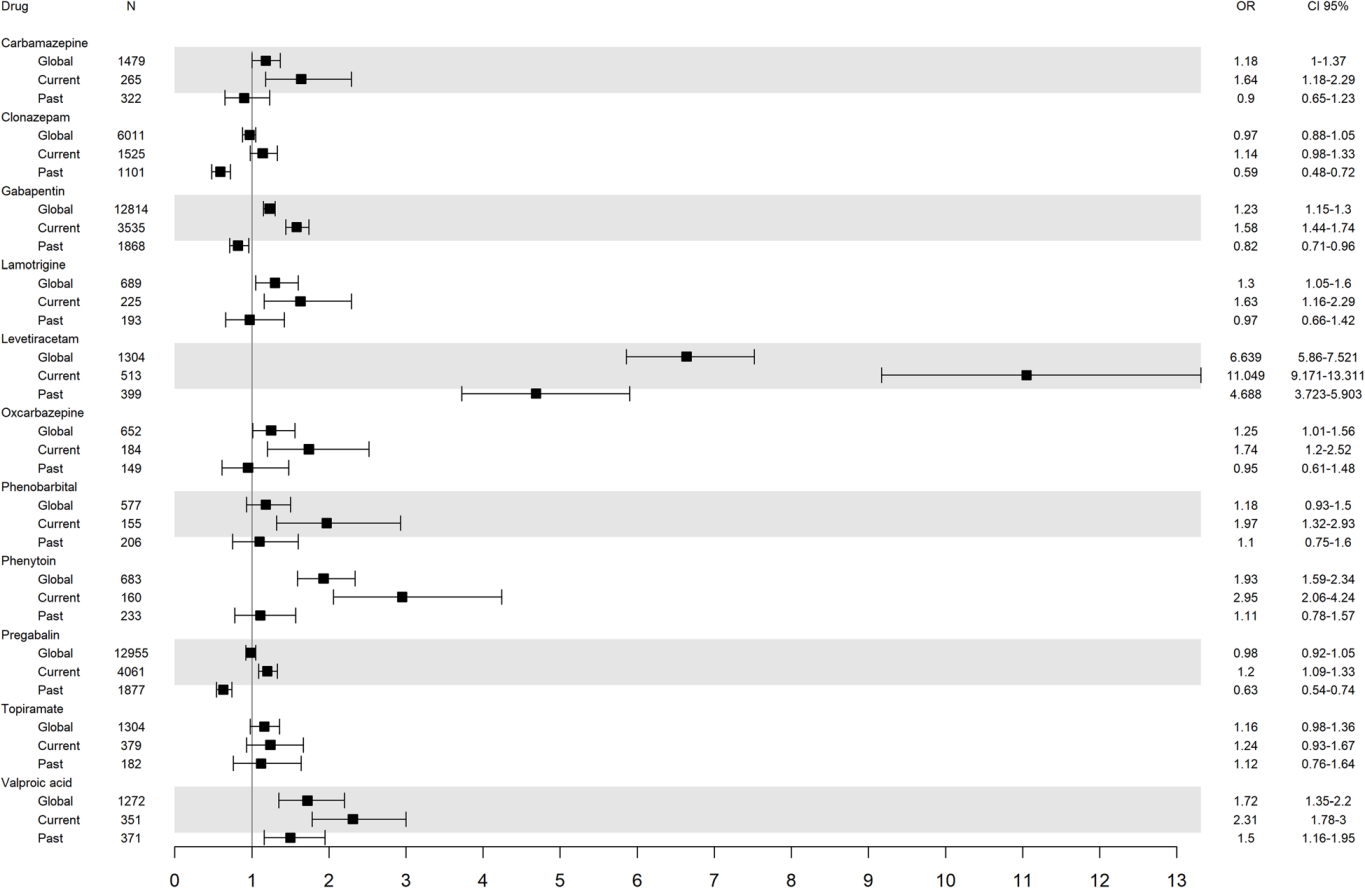
Risk of ischaemic stroke according to different exposures to antiepileptic drugs

The effect of the AED exposure was adjusted for different baseline variables for the regression models (Fig. 2). Global exposure to AED showed a risk of 1.06 (95 % CI 0.99–1.13, *p* = 0.057) of ischaemic stroke and above all, the exposure to levetiracetam [(OR_adj_ 3.3 CI95 % 2.8-4)], phenytoin [(OR_adj_ 1.5 CI95 % 1.2–1.9)] and valproic acid [(OR_adj_ 1.3 CI95 % 1.1–1.6)] did. Past exposure to lamotrigine [(OR_adj_ 1.6 CI95 % 1.5–1.7)], phenobarbital [(OR_adj_ 2.1 CI95 % 1.3–3.2)] and phenytoin [(OR_adj_ 1.6 CI95 % 1-2.5)] showed a risk but only levetiracetam [(OR_adj_ 4.1 CI95 % 3.3–5.2)] and valproic acid [(OR_adj_ 1.4 CI95 % 1.1–1.9)] showed it when assessing current use. When the effect of AED in monotherapy was analysed, only levetiracetam (OR_adj_ 5.1 CI95 % 3.7–6.9) was associated with a high risk of ischaemic stroke.

## Discussion

Most studies assessing risk of stroke with use of AED were cohort studies which did not analyse different times of exposure as in our study, our research analyse global, past, current and current monotherapy exposures through a case-control design. We found that global exposure to AED was not significantly associated to ischaemic stroke (OR 1.06, CI95 % 0.998–1.128) [8–17].

Among the classic AED (phenobarbital, phenytoin, valproic acid, carbamazepine and clonazepam) only the exposure to phenytoin and valproic acid showed a risk for ischaemic stroke that was even higher for current exposure in the case of valproic acid. The highest risk for these classics was for the old phenobarbital past exposure showing an adjusted risk of 2.0 (CI95 % 1.3–3.2). Regarding phenytoin and valproic acid, they demonstrated higher risk of stroke when compared to carbamazepine in the study of Hsieh et al. [16]. A pharmacokinetic explanation, regarding the role as potent inducers of cytochrome P450 enzymes (involved in cholesterol synthesis) of carbamazepine or phenytoin could substantially increase the risk of cardiovascular and cerebrovascular disease [28]. When compared to the new AED, only past exposure to lamotrigine (OR_adj_ 1.6 CI95 % 1.5–1.7) showed a risk for stroke. Renoux et al. analysing the past exposure to AED did not find an increased risk of ischaemic stroke (RR 0.89, CI95 % 0.78–1.01) [15]. In the case of those AED which were associated to an increased risk of stroke for the past exposure but not for the current, we are not able to conclude if this higher risk was caused by the epilepsy itself or by the drug exposure [7, 18, 19, 29].

Nevertheless, measuring current exposure to AED may be more accurate in order to assess the real increase of risk of ischaemic stroke caused by these drugs. In our study, levetiracetam exposure was associated with the highest risk of stroke [OR adj 3.3 (CI95 % 2.8- 4.0)] even higher when assessing monotherapy exposure [OR adj 5.1 (CI95 % 3.7–6.9] was assessed. Levetiracetam shows a risk for stroke when handle the indication bias taken only first incident stroke cases excluding prescriptions in the same month of the index date. To be considered that levetiracetam is used for the most severe and for refractory epilepsies when other AED fail, information not available in our database, what could address a risk of the epilepsy itself. We have not found any studies with similar results for levetiracetam. Deeper studies have to be conducted, analysing the AED indications what could be relevant to support our results.

Clonazepam is a benzodiazepine usually used as an “add-on” medication for people who continue to have seizures while taking other seizure medicines. Not having diagnosis linked to its prescription can refer to its use for psychiatric disorders and not really to prevent seizures. It can also be prescribed as “if needed” which wouldn’t reflect its use.

The AED studied with higher prevalence of use in our population were gabapentin and pregabalin, drugs which have other indications with a more frequent use than epilepsy; both are authorized for neuropathic pain and pregabalin is also authorized to treat anxiety disorders, which are more prevalent disorders than epilepsy. None of these drugs show a risk for stroke for the different exposures estimated.

Among AED those with a mainly use in our country for epilepsy, phenytoin and valproic acid, showed a global risk [OR adj 1.5 (CI95 % 1.2–1.9)] [OR adj 1.34 (CI95 % 1.1–1.6)] which are not maintained in the past exposures meanwhile lamotrigine and phenobarbital showed a risk for the past exposure [OR adj 1.58 (CI95 % 1.5–1.7) and OR adj 2.08 (CI95 % 1.3–3.2) respectively], thus we cannot rule out that the epilepsy itself is increasing stroke risk.

One of the main limitations of our data is the lack of association between a drug prescription and a diagnosis registered, or the lack of registry of the severity of epilepsy. We cannot know the diagnosis which led to the AED prescription, not only seizures but also the possibility of other diagnosis that could lead to AED prescription such as tumour, hypoxia, and neuropathic pain, among others. The main cause of seizures in the elderly is stroke (52.3 %), [30] what can point to an indication bias in our study, we have even tried to handle it by excluding those patients a first stroke episode and excluding those AED prescriptions in the same month of the index date. There are no diagnostic images available in our database, so we were not able to examine a possible ischemic lesion by imaging study in either the controls or the cases, thus, this remains as a limitation inherent to our study.

## Conclusions

Drugs used for other conditions than epilepsy were the most used AED (pregabalin, gabapentin). They didn’t show a risk for stroke.

An inherent risk of the epileptic condition for stroke cannot be dismissed with the present study results, but no link between drugs and diagnosis in our database made this a limitation in our research.

Levetiracetam shows a risk for stroke global exposure, current and also monotherapy.

Even our effort for handling the indication bias the lack of data regarding the diagnosis, severity and kind of epilepsy for it use should be address with further research focus on this active substance.

## Supplementary Information

Additional file 1: **Table S1. **Multivariate model.

## Data Availability

All datasets are available at request to the corresponding author.

## Abbreviations

AED: Antiepileptic drugs
ATC: Anatomic Therapeutic and Chemical
BMI: Body mass index
CI: Confidence interval
CMBD-HA: Minimum Data Set at Hospital Discharge
CNS: Central Nervous System
DALY: Disability-Adjusted Life Year
ECAP: Electronic health records in Primary healthcare
GFR: Glomerular filtration rate
GP: General Practitioner
ICD: International Classification of Diseases
ICS: Catalan Health Institute
MDRD: Modification of diet in renal disease
MEDEA: Socioeconomic index
NSAID: Non-steroidal anti-inflammatory drugs
OR: Odds ratio
PHC: Primary healthcare
SD: Standard deviation
SIDIAP: Information system for research in primary care
