# Supplementary material for: Risk of ischaemic stroke associated with antiepileptic drugs: a population-based case-control study in Catalonia
Source: BMC Neurol. 2021 May 24;21:208. doi: 10.1186/s12883-021-02237-1 (PMC8142644; doi:10.1186/s12883-021-02237-1)
Supplement: Supplementary file 1 — Table S1. Multivariate model. [file 12883_2021_2237_MOESM1_ESM.docx]

## Table S1. Multivariate model

| **N= 22,271** | **OR (CI 95%)** | **p** |
| --- | --- | --- |
| **Age** | 0.99 (0.98-1.00) | <0.001 |
| **Sex (ref. men)** | 1.07 (1.02-1.11) | 0.003 |
| **Year of index date** | 0.93 (0.92-0.94) | <0.001 |
| **N. visits PHC (ref. <5)**  5-9  10-24  ≥25  Unknown | 1.03 (0.97-1.10)  1.38 (1.30-1.48)  2.01 (1.87-2.17)  0.79 (0.72-0.87) | 0.347  <0.001  <0.001  <0.001 |
| **Smoking habit** | 1.60 (1.52-1.70) | <0.001 |
| **BMI (ref. normal)**  Overweight  Obesity  Unknown | 0.84 (0.78-0.91)  0.78 (0.72-0.84)  1.60 (0.96-1.10) | <0.001  <0.001  0.478 |
| **MEDEA (ref. U1)** | 1.00 (0.94-1.07) | 0.898 |
| **GFR (ref. >60 mL/min/1.73m^2^)**  45-60  30-44  <30  Unknown | 1.16 (1.09-1.24)  1.26 (1.15-1.37)  1.56 (1.36-1.78)  1.03 (0.98-1.08) | <0.001  <0.001  <0.001  0.247 |
| **Arthrosis** | 0.92 (0.87-0.96) | <0.001 |
| **Dementia** | 1.37 (1.24-1.51) | <0.001 |
| **Depression** | 1.02 (0.94-1.10) | 0.680 |
| **Diabetes** | 1.11 (1.04-1.19) | 0.003 |
| **Dyslipidaemia** | 0.69 (0.66-0.72) | <0.001 |
| **Epilepsy** | 1.71 (1.44-2.02) | <0.001 |
| **Fibromyalgia** | 0.89 (0.73-1.08) | 0.248 |
| **Gastrointestinal ulcer** | 1.06 (0.95-1.17) | 0.308 |
| **Ischemic heart disease** | 0.88 (0.82-0.94) | <0.001 |
| **Hypertension** | 0.95 (0.90-1.00) | 0.041 |
| **Neuropathies** | 0.95 (0.87-1.05) | 0.333 |
| **Peripheral artery disease** | 1.97 (1.80-2.14) | <0.001 |
| **Acetic acid derivatives** | 0.99 (0.95-1.03) | 0.530 |
| **Analgesics (metamizol and paracetamol)** | 1.08 (1.02-1.15) | 0.011 |
| **Antihypertensives** | 1.14 (1.03-1.25) | <0.001 |
| **Beta-blockers** | 1.41 (1.33-1.48) | <0.001 |
| **Calcium channel-blockers** | 1.17 (1.11-1.23) | <0.001 |
| **Blood glucose-lowering drugs** | 1.07 (0.99-1.15) | 0.068 |
| **Cardiac therapy** | 1.33 (1.25-1.41) | <0.001 |
| **Coxibs and Oxicams** | 0.88 (0.84-0.93) | <0.001 |
| **Diuretics** | 1.16 (1.11-1.21) | <0.001 |
| **Gastrointestinal tract** | 1.28 (1.14-1.43) | <0.001 |
| **Insulins** | 1.34 (1.24-1.44) | <0.001 |
| **Lipid-modifying agents** | 2.04 (1.95-2.14) | <0.001 |
| **Opioids (phentanil, buprenorphine and tramadol)** | 1.04 (0.99-1.09) | 0.122 |
| **Other anti-inflammatories (glucosamine and chondroitin sulphate)** | 0.87 (0.82-0.93) | <0.001 |
| **Propionic acid derivatives** | 0.95 (0.91-1.00) | 0.041 |
| **Renin-angiotensin agents** | 1.33 (1.27-1.40) | <0.001 |
| **Current Use**  Anti-dementia agents  Antidepressants  Antipsychotics  Anxiolytics  Psychostimulants | 0.77 (0.68-0.88)  1.17 (1.10-1.23)  1.59 (1.47-1.73)  1.17 (1.12-1.23)  1.54 (1.35-1.76) | <0.001  <0.001  <0.001  <0.001  <0.001 |

*OR, odds ratio. CI, confidence interval. PHC, primary healthcare. BMI, body mass index. MEDEA, socioeconomic i
